# The complete chloroplast genome sequence of plumed cockscomb (*Celosia argentea*, Amaranthaceae)

**DOI:** 10.1080/23802359.2019.1623128

**Published:** 2019-07-10

**Authors:** Ying-Xi Qian, Jing Gao, Yong-Hui Jin, Rui-Hong Wang, Ling Xu, Zhe-Chen Qi

**Affiliations:** a Zhejiang Province Key Laboratory of Plant Secondary Metabolism and Regulation, College of Life Sciences and Medicine, Zhejiang Sci-Tech University, Hangzhou, China;; b Shanghai Key Laboratory of Plant Functional Genomics and Resources, Shanghai Chenshan Botanical Garden, Shanghai, China

**Keywords:** *Celosia argentea*, chloroplast genome, phylogenomics, plumed cockscomb

## Abstract

The complete chloroplast genome of *Celosia argentea*, an important horticultural and medicinal herb, was identified and sequenced in this study. The genome size is 153,474 bp, the GC content is 36.7%. A total of 123 genes were identified, including 84 protein-coding genes, 8 rRNA genes, and 33 tRNA genes. Twenty-nine plastome accessions from Caryophyllales were selected to assess the phylogenetic placement of genus and the result showed that *C. argentea* is most closely related to *Amaranthus hypochondriacus*.

*Celosia argentea*, Amaranthaceae, is an annual herb characterized by lanceolate leaves and prominent terminal inflorescences. Over 100 cultivars of *C. argentea* and its variety var. *cristata* has been widely grown in most tropical and subtropical countries around the world (Nath et al. 1997). It is popularly known as cockscomb due to its comb-shaped inflorescences. Additionally, it has been used in herbal medicine due to its antivirus, diuretic, and hypotensive properties (Tolouei et al. 2018). Previous genetic study of *C. argentea* using SRAP showed a high level of diversity at the population level (Feng et al. 2009). However, no chloroplast genome resource is available so far for this economically important herb. In this study, the first complete chloroplast genome of *Celosia* is reported.

The sample was collected from Hangzhou, Zhejiang, China (E120°01′39″, N30°06′23″, Voucher No. ZSTU00833, deposited at Zhejiang Sci-Tech University). Total genomic DNA was extracted from fresh mature leaves of *C. argentea* individual using DNA Plantzol Reagent (Invitrogen, Carlsbad, CA, USA). The plastome sequences were generated using Illumina HiSeq 2500 platform (Illumina Inc., San Diego, CA, USA). The CLC *de novo* assembler (CLC Bio, Aarhus, Denmark), BLAST, GeSeq (Tillich et al. 2017), and tRNAscan-SE v1.3.1 were used to align, assemble, and annotate the plastome (Peter et al. 2005).

The full length of *C. argentea* chloroplast genome (GenBank Accession No. MK598853) was 153,474 bp. It is made up of a large single-copy region (LSC with 84,842 bp), a small single-copy region (SSC with 21,166 bp) and two inverted repeat regions (IRs with 23,733 bp). Total GC content is 36.7%. A total of 123 genes are successfully annotated, including 84 protein-coding genes, 33 tRNA genes, and 8 rRNA genes. The tRNA genes are distributed throughout the whole genome with 21 in the LSC, three in the SSC, and nine in the IR regions, while rRNAs are only situated in the IR regions. The content of protein-coding genes, tRNA genes, and rRNA genes are 68.3%, 26.8%, and 6.5%, respectively. Seven genes of tRNA (*trnM-CAT, trnI-CAT, trnL-CAA, trnV-GAC, trnI-GAT, trnN-GTT, trnR-ACG,* and *trnL-TAG*) had two copies and all four rRNA species (*rrn4.5*, *rrn5*, *rrn16*, and *rrn23*) also had two copies. Among the protein-coding genes, two genes (*ycf3* and *clpP*) contained two introns, and other four genes (*atpF, ropC1, ndhB,* and *ndhA*) had one intron each.

Using MAFFT v7.3 (Kazutaka and Standley 2013), we aligned 29 chloroplast genomes of species from Caryophyllales. A phylogenetic tree was drawn by statistical method of the maximum likelihood (ML) inference using GTR + G model with 1000 bootstrap replicates with RAxML v.8.2.1 (Alexandros 2014) on the CIPRES cluster service (Miller et al. 2010). The result showed that *C. argentea* is closely related to *Amaranthus hypochondriacus* (Figure 1). This newly reported chloroplast genome will provide valuable information for genetic evolution and molecular breading studies of *Celosia*.

**Figure 1.**
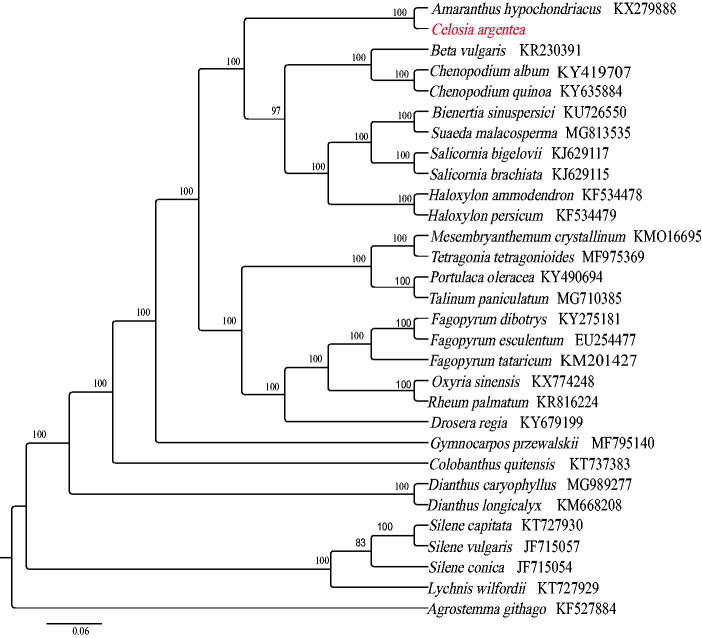
The best maximum likelihood (ML) phylogram inferred from 30 chloroplast genomes in Amaranthaceae and Caryophyllales.
